# Experimental Evaluation of Anion Exchange Membranes for the Desalination of (Waste) Water Produced after Polymer-Flooding

**DOI:** 10.3390/membranes10110352

**Published:** 2020-11-18

**Authors:** Paulina A. Sosa-Fernández, Jan W. Post, Harrison L. Nabaala, Harry Bruning, Huub Rijnaarts

**Affiliations:** 1 European Centre of Excellence for Sustainable Water Technology, Wetsus, P.O. Box 1113, 8911CC Leeuwarden, The Netherlands; paulina.sosafernandez@wetsus.nl (P.A.S.-F.); jan.post@wetsus.nl (J.W.P.); nleshao88@gmail.com (H.L.N.); 2 Department of Environmental Technology, Wageningen University, P.O. Box 8129, 6700EV Wageningen, The Netherlands; huub.rijnaarts@wur.nl

**Keywords:** anion-exchange membranes, produced water, electrodialysis

## Abstract

Electrodialysis (ED) has been recently proposed to desalinate polymer-flooding produced water (PFPW), a byproduct stream from the oil and gas industry rich in charged polymers. However, process performance is limited by fouling occurring on the ion-exchange membranes, particularly on the anionic ones (AEMs). Thus, this study aimed to correlate the properties of different AEMs with their performance while desalinating PFPW, ultimately evaluating their significance when fouling is to be minimized and operation improved. Six stacks containing different homogeneous and commercially available AEMs were employed to desalinate synthetic PFPW during 8-days ED experiments operated in reversal mode. AEMs recovered from the stacks were analyzed in terms of water uptake, ion-exchange capacity, permselectivity, and area resistance, and compared with virgin AEMs. Relatively small changes were measured for most of the parameters evaluated. For most AEMs, the water uptake and resistance increased, while the ion-exchange capacity (IEC) and permselectivity decreased during operation. Ultimately, AEMs with high area resistance were linked to the fast development of limiting current conditions in the stack, so this property turned out to be the most relevant when desalinating PFPW.

## 1. Introduction

Electrodialysis (ED) is a versatile electro-membrane process capable of removing ions and small charged particles from a stream by applying an electric potential over an arrangement of ion-exchange membranes (IEMs) [1,2]. One of its most recent applications is the treatment of polymer-flooding produced water (PFPW), an abundant stream from the oil and gas industry obtained after applying polymer flooding technology [3,4,5]. After reducing its salinity, PFPW could be reused to confect viscous flooding solution without the need to add excessive amounts of viscosifying polymer and chemicals, increasing chemical use efficiency, thus leading to environmental and economic benefits [6,7]. However, membrane fouling remains a recurrent issue that needs to be tackled to expedite the application of ED at an industrial scale [8,9].

Fouling designates the undesirable attachment of species on the surface or the inner part of a membrane [10]. Fouling can appear as a consequence of concentration polarization, for example, due to the accumulation and precipitation of salts (scaling), or triggered by the local changes in pH when water dissociates on the surface of the IEMs [11]. Ultimately, fouling causes alterations in the membrane structure, decreased membrane permselectivity, water dissociation, and reduced process performance [10]. These complications also affect the desalination of PFPW, making fouling a critical issue to be resolved when treating such industrial water streams [12,13,14]. Since PFPW contains a mixture of organic compounds, dissolved gases, solid impurities, and minerals [15], fouling can be easily formed on the IEMs. The positively charged anion exchange membranes (AEMs) are especially prone to be fouled by negatively charged organic molecules, like partially hydrolyzed polyacrylamide (HPAM) [13,16,17], which is the most commonly employed viscosifier in polymer-flooding oil and gas reservoirs under exploitation [18].

Although the fouling nature of the feed solution is difficult to amend, there exist several strategies to control fouling during ED. A fundamental approach is the control of the hydrodynamic conditions to ensure the adequate mixing of the streams, and thus minimize concentration polarization. Adequate mixing is generally achieved by pumping at high flowrates, employing spacers designed to increase the turbulence, and by sparging air or other gases into the stack [19]. Besides, there are other strategies to prevent and control fouling during ED: application of electrodialysis with polarity reversal (EDR), incorporation of pretreatments, use of pulsed electric field, modification of the membranes, and the design of specific cleaning routines [10,20]. In EDR, the concentrating and diluting compartments are alternatively switched by periodically reversing the direction of the electric current [15,21]. In this way, ions for which the membranes are permeable pass through the membranes moving in the opposite direction, while impurities adsorbed on the membranes are removed. However, one of the main disadvantages of EDR is the reduced efficiency due to the streams mixing every time that they are switched. Many of the other strategies have already been tested for desalinating PFPW. Ultrafiltration (UF) has been incorporated as a standard pretreatment since it reduced the severity of fouling on the IEMs. However, fouling still occurs since UF does not eliminate all the organic components present in the stream [16,22,23]. Cleaning routines have also been tested and optimized to remove the particular fouling caused by PFPW [12,14]. However, their use can also lead to potential negative impacts on membrane selectivity and performance [10,24].

Another of the strategies, the application of pulsed electric field (PEF), was recently tested by our research group for desalinating PFPW [25]. Besides concluding that concentration polarization caused most of the decreases in performance, it was surprising to observe that the fouling on the IEMs was minimal, especially considering our previous studies [6,13]. These observations included membranes utilized to desalinate PFPW with HPAM and crude oil in the continuous mode. Two reasons could explain the results: (i) the duration of the experiments (2–6 h) was too short for producing significant fouling, and (ii) the type of membranes employed (FujiFilm-10, based on an aliphatic matrix) also influenced the results.

In this line, recent investigations also suggest that the chemistry of the membranes and its water content are the main factors influencing the fouling of organic compounds [26]. When studying fouling by PFPW on PVDF membranes, Zuo et al. explained the antifouling characteristics of SiO_2_/PVDF AEMs in terms of their hydrophilicity. They described that the mixture SiO_2_/PVDF was more hydrophilic than PVDF alone, which allowed these membranes to interact stronger with the water molecules than with the organic and oily components in the feed [27]. Additionally, while performing electrodialysis on PFPW, Wang et al. concluded that the fouling on heterogeneous IEMs was more severe than the one on homogeneous IEMs due to physical blockage [16].

As presented, there are various indications that AEMs susceptibility to fouling, when desalting PFPW, relates to material and matrix characteristics. Besides water content, it is desired to know which other membrane characteristics can be correlated to a high or low fouling incidence by treating PFPW. Thus, the general objective of this study is to determine which AEM properties are beneficial in terms of minimizing the effects of fouling and improving the performance when employed to desalinate PFPW. The research is focused on six commercially available homogeneous AEMs, which were employed to desalinate PFPW during relatively long term experiments. To extend the process lifetime and increase membrane exposure, the EDR operation mode was employed, as it would be commonly used when desalinating industrial water. Then, the membranes were recovered, and their properties evaluated. The results were compared against the properties measured for virgin membranes, and for membranes soaked in the feed solutions without the application of an electric potential (sorption experiments), aiming to improve the understanding of the role of membrane material on fouling.

## 2. Materials and Methods

Experiments were planned to evaluate the performance of six different kinds of AEMs during long term electrodialysis runs with polarity reversal. After the ED experiments, the membranes were recovered from the stack, and their properties analyzed.

### 2.1. Materials

#### 2.1.1. Preparation of Solutions

The composition of the synthetic feed solution employed for all experiments was based on that of the brackish water (BW) in the Marmul field in Oman [28], as outlined in Table 1. All the salts employed (NaCl, KCl, MgCl_2_.6H_2_O, CaCl_2_.2H_2_O, Na_2_SO_4_, and NaHCO_3_) were analytical grade, purchased from VWR (Leuven, Belgium), and used without further purification.

Synthetic polymer-flooding produced water was prepared by adding the necessary amount of partially hydrolyzed polyacrylamide (HPAM) to 10.0 L of BW solution under fast stirring (≈750 rpm). After pouring the HPAM, the stirring rate was reduced to 250 rpm, and the solution was left mixing overnight to assure complete HPAM hydration. The resulting solution is referred to as brackish water + polymer (BW + P) solution.

During the last part of the ED experiments, the feed PFPW contained emulsified crude oil. To prepare the emulsion, 2.0 g of crude oil was added to 2.0 L of BW previously heated to 45 °C. The emulsification was done with an emulsifying mixer set at 15,000 rpm for 15 min. This stock solution was used to obtain 20 and 40 mg/L of oil emulsions employed in the 10 L feed solution, also referred to as BW + P + O solution. The stock solution was immediately added to the feed to avoid separation of the non-soluble part when left unused for long periods.

The HPAM employed was AB-305-VLM (MW = 600–900 kDa and 30% hydrolyzed), kindly provided by SNF Floerger (Andrezieux, France). The crude oil originated from the North Sea and was provided by Shell. It must be added that this crude oil was used due to availability, but its properties might differ from those of the crude oil from Marmul [29].

#### 2.1.2. Ion Exchange Membranes

For this study, six different types of homogeneous anion exchange membranes were used. All membranes contain quaternary ammonium anion exchange groups and report high permselectivity. FujiFilm-1 and FujiFilm-10 from were selected based on their chemistry. The following membranes were either purchased or provided by the industry: Neosepta AMX from Eurodia (France); FujiFilm types 1, 10, and 12 by FujiFilm Manufacturing B.V. (Tilburg, The Netherlands); Suez AR204 E and Suez AR908 E by Suez Water Technologies and Solutions (Ontario, Canada).

The CEM employed was Neosepta CMX, a membrane that contains sulfonic acid groups as fixed charges. Its properties have been previously reported in the literature [30,31].

#### 2.1.3. Electrodialysis Setup

Experiments were performed using a cross-flow ED stack composed of five cell pairs. The stack contained six Neosepta CMX membranes plus five AEMs of the same type, as listed in Table 2. All membranes employed to assemble a stack were new, with an active membrane area of 0.01 m^2^. The membranes were separated by 485 µm polyamide woven spacers (Sefar 06-700/53, Heiden, Switzerland) with coated silicon rubber at the sides as gaskets (Aquabattery, the Netherlands). The cell contained electrodes made of titanium with mixed metal oxide coating from Magneto Special Anodes BV (Schiedam, the Netherlands). The housing of the stack was kindly provided by REDstack BV (Sneek, the Netherlands). It consisted of polymethyl methacrylate side plates for the water distribution and end plates with the electrode compartments, plus the materials needed to close the assembly. A scheme of the stack is available in [19].

The electrical current was controlled by a potentiostat/galvanostat Ivium-n-Stat (Ivium Technologies, Eindhoven, The Netherlands). The potential difference over the membrane stack was measured using two reference Ag/AgCl gel electrodes (QM711X, QIS, Roosendaal, The Netherlands) placed at the inlet of each electrode compartment and connected to the Ivium-n-Stat.

The feed and electrolyte solutions were pumped by three independent peristaltic pumps from Cole-Parmer, Masterflex L/S Digital drive (Barendrecht, The Netherlands). The conductivities of the diluate and the concentrate streams exiting the ED stack were continuously measured with conductivity probes (Orion DuraProbe 4-electrode conductivity cell 013005MD). A transmitter box (Orion Versastar Pro) connected to the probes corrected the measurements to the reference value at 25 °C and recorded them on the computer. The pH of the diluate and concentrate was also continuously measured by two pH probes (MemoSENS Endress + Hauser, pH range 1 to 12) placed after the ED stack. The pH probes were connected to a transmitter box (P862, QIS, Endress + Hauser). Two back-pressure valves, set at 0.25 bar, were placed at the outlet of electrolyte solution to ensure it was evenly distributed through the electrodes.

### 2.2. Methods for the EDR Runs

#### 2.2.1. Electrodialysis Reversal Runs

**Figure 1:**
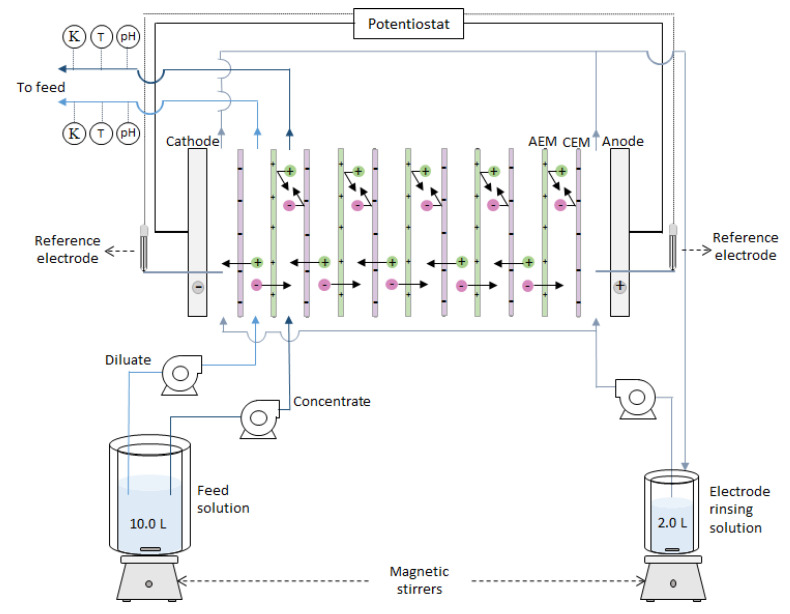
Scheme of the setup employed. The experiments were run in a batch recirculation mode, in which two streams were taken from the same feed solution, circulated through the ED-stack, and returned to the same reservoir. Adapted from [6].

EDR experiments were conducted at a constant current in a batch recirculation mode, meaning that the diluate and concentrate streams were both fed from the same vessel, went through the ED stack, and returned to the feed container (Figure 1). The vessel contained 10.0 L of the feed solution, which composition and flowrate were varied for the different stages of the experiment, as specified in Table 3. Since the first stack assessed was the one containing Neosepta AMX, some operational parameters were determined based on its performance and later implemented on the subsequent experiments. For example, the switch to solution III (BW + 50 mg/L HPAM) was done together with an increase in linear velocity to avoid operating at high voltages, and it was further increased when switching to stage IV (BW + 100 mg/L HPAM). Since there was a rapid electric potential increase when stage V (BW + 200 mg/L HPAM) started, the current density was reduced to 75 A/m^2^.

Overall, the EDR was run at a constant current between 75 and 100 A/m^2^ for nearly 200 h (as described in Table 3) with current reversal every hour (the lower current density was applied to avoid reaching the 10 V limit of the potentiostat). The electrolyte solution, consisting of 0.14 M Na_2_SO_4_, was circulated at 170 mL/min and was replaced simultaneously with the feed solutions. The experiments were performed in a laboratory with controlled temperature at 23 ± 1 °C.

Each experiment started by running the electrodialysis for at least 12 h with 0.5 M NaCl to ensure the optimum assembly of the stack. Then, the solution was substituted by BW, and the stack performance was measured to confirm that 100 A/m^2^ could be supplied without reaching limiting current density (LCD) conditions.

During the experiments, the applied current, stack voltage, conductivity, pH, and temperature were monitored continuously. Samples from all streams were periodically taken. After each experiment, the stack was disassembled, and all the membranes recovered for analysis.

#### 2.2.2. Degree of Demineralization

The degree of demineralization (*D_dem_*, %) was calculated employing the initial conductivity of the feed solution *c*_0_ (mS/cm) and the conductivity of the diluate solution *c_d_* (mS/cm), according to Equation (1) [33].

(1) $$D_{dem}=\;\;\frac{C_{0}-C_{d}}{C_{0}}\times 100$$

### 2.3. Methods for Membrane Characterization

#### 2.3.1. Water Uptake and Hydration Number

The water uptake (WU), also called swelling degree by some authors, indicates how the membrane mass changes when exposed to water [34]. For its determination, wet membrane coupons were removed from 0.5 M NaCl solution, their outer surface was smoothly wiped with a laboratory tissue, and their wet mass (*m_w_*) recorded. Then, the coupons were placed in Petri dishes and left in a vacuum oven at 30 °C to dry overnight. Then, the dry mass (*m_d_*) of the coupons was measured, and the *WU* calculated as the mass of removed water divided by the dry mass [35,36]:

(2) $$WU=\frac{m_{w}-m_{d}}{m_{d}}$$

The hydration number (*λ*) [37] or water content [34] of the membranes was calculated by dividing the *WU* by the molecular weight of water and the ion exchange capacity (IEC, meq/g-dry) of the membrane. A factor of 1000 is necessary to report *λ* in mol H_2_O/mol counterion.

(3) $$\lambda =\frac{WU\times 1000}{MW_{H_{2}O}\times IEC}$$

#### 2.3.2. Ion-Exchange Capacity (IEC), Charge Density, and Charge per Area

The ion-exchange capacity (IEC) of a membrane reports the number of ion-exchange equivalents per mass of dry membrane (meq/g-dry). It was determined by exchanging their counterion from chloride to nitrate and then measuring the amount of chloride released. First, AEM coupons were conditioned in 0.5 M NaCl solution for at least 48 h. Then, they were rinsed with Milli-Q water (to ensure that excess chloride on the surface was removed) and soaked in 200 mL of 0.5 M NaNO_3_ while stirring at 300 rpm. In this way, the Cl^−^ in the membrane is exchanged by NO_3_^−^. After 24 h, samples of the solution were taken and analyzed for their chloride content via ion chromatography (see Section 2.3.7). This concentration is proportional to the number of ionic charges present in the membrane sample, so once obtained, it was converted in meq and divided by the *m_d_* to obtain the IEC [26,31,38].

The charge density (meq/g H_2_O), which reflects the equivalents for the water-swollen membrane, was calculated by dividing the measured IEC by the *WU* [39].

#### 2.3.3. Permselectivity

The permselectivity (α) of the AEMs was experimentally obtained by dividing the measured potential (Δ*U_meas_*) over the theoretical one (Δ*U_theo_*) for a 100% permselective membrane (Equation (4)), the last calculated through the Nernst Equation. The activity coefficients of the 0.5 M and 0.1 M NaCl solutions were considered as 0.686 and 0.778, respectively [31].

(4) $$\alpha =\frac{\Delta U_{meas}}{\Delta U_{theo}}$$

Δ*U_meas_* was determined by placing a membrane coupon in the middle of a two-compartment cell (Figure S1). Two solutions, 0.1 M and 0.5 M NaCl were circulated through each compartment of the cell at 300 mL/min. Both solutions were maintained at 25 °C utilizing a recirculation bath. The potential difference across the membrane was measured using two Ag/AgCl reference electrodes, each placed inside one compartment, and connected to the Ivium potentiostat/galvanostat. The potentiostat was set in the open cell mode to register the potential generated from the sample membranes. The potential registered within the last 15 min of a running system was averaged to obtain the mean value after the system had stabilized.

#### 2.3.4. Electrical Resistance

The electrical resistance of the AEMs was measured in a six-compartment cell, formerly described [40]. The four inner compartments contained either 0.5 M NaCl or BW solution, while the two outermost ones had 0.5 M Na_2_ SO_4_ as the electrolyte solution. All solutions were pumped at 170 mL/min using peristaltic pumps. The active area of the membrane under evaluation was 7.07 cm^2^. The potential drop across the membrane was measured by two Habber–Luggin capillaries, each placed on one side of the membrane, filled with 3.0 M KCl solution, and connected to an Ag/AgCl gel reference electrode. The measurements were performed at 25 °C.

The measurements were performed on the same membrane coupons previously tested for permselectivity, which were conditioned in 0.5 M NaCl. The area resistance was determined using chronopotentiometry: constant current densities were applied for 2 min each, while the electric potential was recorded. The current was controlled by Autolab PGstat12 (Utrecht, The Netherlands). Each type of membrane was measured three times, and additional measurement was performed without membrane to obtain a blank. The area resistances (Ω cm^2^), with and without membrane, were taken as the slope of the plot with the applied current density *i* (A/cm^2^) on the *x*-axis and the potential *U* (V) on the *y*-axis. Then, as shown in Equation (5), the membrane resistance *r_m_* was calculated by subtracting the resistance of the blank *r_s_* from the resistance with the membrane *r_m+s_*.

(5) $$r_{m}=r_{m+s}-r_{s}$$

#### 2.3.5. Single AEM Characterization in Six-Compartment Cell

The six-compartment cell was also employed to characterize the behavior of single AEMs when employed to desalinate the BW + P solution. The cell had the same configuration as previously described, with compartments the four inner compartments filled with the BW solution. The AEM under study was placed in the middle of the cell and employed to desalinate the solution in compartment four at a constant current density of 28 A/m^2^ [17]. After 30 min, the solution running through compartments 3 and 4 was substituted by BW + P solution containing 200 mg/L HPAM, after which the desalination continued for 90 min.

#### 2.3.6. Sorption Tests

Sorption tests were performed to evaluate the effect of exposing the membrane to HPAM-containing solutions, but without passing a current through them. The tests were performed by dipping virgin membrane coupons in 0.5 L of BW + P solutions containing 200 mg/L HPAM. The solution was contained in plastic boxes that were kept closed and stored at lab temperature. After 48 h, the membranes were recovered for analysis, and samples of the solution were collected and analyzed for total organic carbon (TOC). The concentration of HPAM was related to TOC measurements through a standard curve (Figure S2).

#### 2.3.7. Solution Analysis

The samples taken during the ED runs and the membrane characterization were analyzed for their ionic and carbon composition. Cations were measured via inductive-coupled plasma optical emission spectroscopy (ICP-OES, Optima 5300DV, Perkin Elmer, Waltham (HQ), MA, USA), and anions through ion chromatography (IC, 761 Compact IC, Metrohm, Tampa, FL, USA). A TOC analyzer (Shimadzu TOC-VCPH) was used to measure total carbon (TC), inorganic carbon (IC), and total organic carbon (TOC).

#### 2.3.8. Electrodialysis Model

The potential U required during the electrodialysis was calculated by modeling the system as an electric DC circuit with resistors in series. The area-specific resistance of a single cell-pair consists of four terms: the area-specific resistance of a CEM (rCEM), that of the compartment filled with a spacer and a concentrated salt solution, that of the AEM (rAEM), and that of the compartment filled with a spacer and a diluted salt solution. Since the feed solution was the same for both compartments and D_dem_ was relatively low, the resistances of the diluate and concentrate solutions can be calculated using the feed conductivity (*c*_0_), so the potential required by the stack can be calculated using Equation (6):

(6) $$U=I\cdot A_{m}\left[\frac{\mathrm{rCEM}}{1-\beta }+N\cdot \left(\frac{\mathrm{rCEM}}{1-\beta }+2\cdot \frac{h}{c_{0}\cdot \left(1-V_{sp}\right)}+\frac{\mathrm{rAEM}}{1-\beta }\right)\right]$$

where *I* is the current (A), *A_m_* is the membrane area (cm^2^), *N* is the number of cell pairs, *β* is the shadow factor, and *V_sp_* is the volume fraction of the spacer [41]. The equation considers the *N* number of cell pairs plus the extra CEM needed to close the stack.

## 3. Results

The first part of this section contains the results from the electrodialysis runs performed with the different AEMs. The second one includes the characterization of the membranes, including the virgin ones, and the ones recovered after the ED and the sorption experiments.

### 3.1. Desalination Performance with Different AEMs

**Figure 2:**
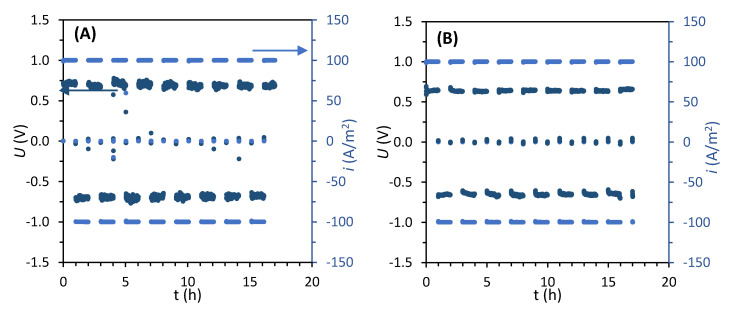
Electric potential *U* (marine) and current density *i* (light blue) versus time during the desalination of NaCl 0.5 M (stage I) in EDR mode. Values measured for the stack composed by (**A**) Neosepta AMX and (**B**) FujiFilm-10. The plots obtained from the other stacks can be found in the Supplementary Material.

After assembling each ED stack, its robustness was tested by desalinating 0.5 M NaCl solution at 100 A/m^2^ for approximately 16 h (stage I). An example of the plots recorded during this stage is included in Figure 2, and the average absolute potential *Ū* recorded for each stack is summarized in Table 4. The lowest electric potential, and therefore the lowest stack resistance, was measured for the stack containing FujiFilm-1 AEMs, which was approximately 72% lower than for the stack with FujiFilm-12 AEMs, the one that registered the highest potential.

Next, the feed solution was switched to BW, and the stack rinsed for 1 h. For the stack containing Neosepta AMX, the LCD was determined to be 180 A/m^2^, corresponding to the inflexion point in the plot of current density versus stack voltage (Figure S4). For the rest of the stacks, a similar method was employed, but the measurement was stopped after ensuring that operating at 100 A/m^2^ would still fall within the ohmic (linear) region. The only stack whose LCD was below 100 A/m^2^ was the one containing FujiFilm-12.

**Figure 3:**
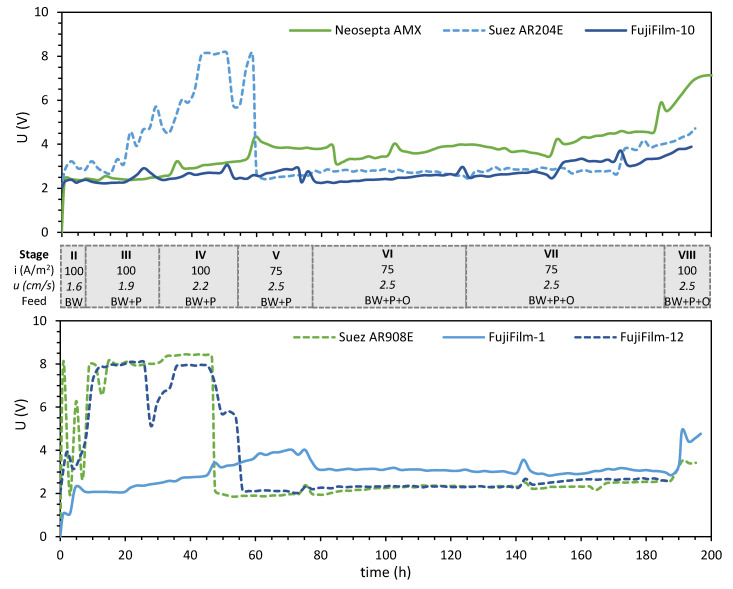
Electric potential *U* versus time during the EDR runs with stacks containing different AEMs. Chronologically, the experiments were performed in the following order: Neosepta AMX, Suez AR204E, FujiFilm-10, Suez AR908E, FujiFilm-1, and FujiFilm-12.

After the LCD tests, the stacks were employed for 6 h to desalinate BW at 100 A/m^2^ (stage II), as shown in Figure 3. Stages III and IV implied increases of the flow velocity, while in stage V the current density was decreased to 75 A/m^2^ to avoid operating at high voltages. The figure shows that when crude oil was incorporated in stage VI no sharp increase in voltage was observed, the first time this occurred during the EDR runs. The relatively stable voltage profile was also observed during stage VII, whose feed solution contained 40 mg/L of crude oil. This stability allowed to switch back to a higher current density (100 A/m^2^) for stage VIII.

Figure 3 also shows that the measured potential fluctuated differently for each of the stacks. Still, it is possible to identify that the membranes Suez AR204E, Suez AR908E, and FujiFilm-12 coincide in reaching higher voltages during the first hours of the experiments when desalinating at a current density of 100 A/m^2^. The figure also shows that for the stages operated at 75 A/m^2^, the voltage remained relatively stable, although slight increases occurred during stage VII for the Neosepta, FujiFilm 10, and Suez AR204 stacks.

The effects of multivalent ions were also observed in two of the stacks. First, the stack containing Suez AR908E presented some voltage peaks after changing from NaCl to BW solution, which was likely related to the presence of multivalent ions. This effect was further analyzed with the FujiFilm 1 stack since the first 4 h of stage II desalinated a BW solution without divalent ions (Ca and Mg). As can be seen in Figure 3, this had a significant effect, since the recorded voltage was nearly half of that needed when desalting BW solution with all its components.

**Figure 4:**
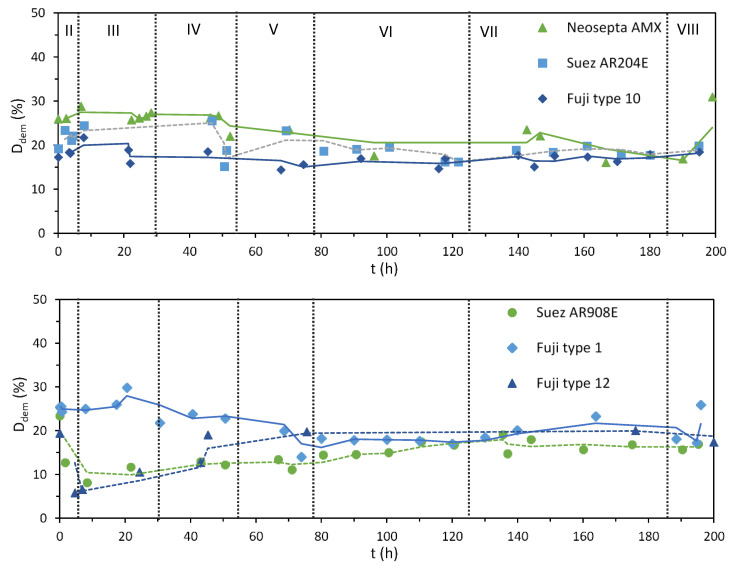
Demineralization after a single pass (%) vs. time during the EDR runs performed with different AEMs. Calculated values are represented by the symbols, and continuous trendlines (calculated as moving average) are included only for guiding the eye. The change of stage is represented by the vertical lines.

Figure 4 shows the degree of demineralization (*D_dem_*) calculated from the measured conductivities of the solutions, according to Equation (1). In general, the degree of desalination achieved with all the stacks was similar, as expected, given the operation at constant current, with values ranging between 10% and 30%. For the stacks composed by Neosepta AMX and FujiFilm-1, the initial *D_dem_* was slightly higher than for the rest, but it leveled at 20% around hour 60, when the current density was lowered to 75 A/m^2^ (stage V). The lower plot also shows the moment when limiting current conditions were reached for the Suez AR908E and the FujiFilm-12 stacks at hour 6, when stage III started, as indicated by their lower demineralization performance.

**Figure 5:**
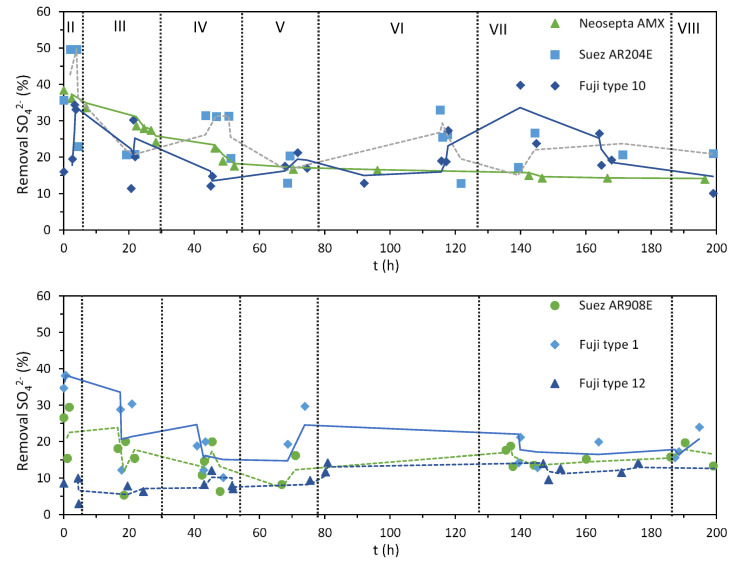
Measured sulfate removal vs. time for each stack. Each data point was calculated from the average changes in sulfate concentration in both the diluate and the concentrate stream. Continuous trendlines (calculated as moving average) are included only for guiding the eye.

Among the anions present in the feed solution, the removal of sulfate is particularly interesting because it is the only divalent, and it is usually transported with more difficulty than the monovalent ones [42]. Figure 5 shows the sulfate removal, which was calculated from the diluate and concentrate samples taken during the runs. The initial SO_4_^2−^ removal values of the stacks varied significantly, from almost 50% for Suez AR204E to 10% for FujiFilm-12, with most values around 30%. The SO_4_^2−^ removal above 30% in the initial stage is expected since this anion was not present in the solution before, so the membranes are still conditioning in the BW solution. Once the membranes are conditioned, the SO_4_^2−^ removal further stabilizes.

### 3.2. Evaluation of the Anion Exchange Membranes

Membrane properties (thickness, water uptake, IEC, permselectivity, and area resistance) were determined for virgin and fouled AEMs. There were two kinds of fouled membranes: the ones recovered from the ED-stack (photographs of these membranes are included in Figure S5), and the ones recovered from the sorption tests. The sorption experiments were performed to assess if the changes measured were caused by PFPW being transported, or if similar behaviors were observed when the AEMs were soaked in BW + P solution. We refer to sorption and not to adsorption tests because, although it has been noticed that the HPAM molecule is too big to go through the IEMs [6], it is possible that segments of the linear chains of the polymer can get inside the membranes.

The properties measured for the virgin membranes are included in Table 5, together with some values reported in the literature.

#### 3.2.1. Physico-Chemical Characterization of Virgin Membranes

In general, the measured **thicknesses** correspond with the ones reported in the literature and by the membrane suppliers (Table 2). The only exception was Suez AR204E, which was over 60 µm thicker than reported by the supplier. Regarding the **IEC** of the AEMs, it ranged between 1.3 meq/g-dry for Neosepta AMX and 2.19 meq/g-dry for FujiFilm-1. For the membranes for which data is available, the measured values do not deviate significantly from other values reported.

Water content is a function of the molar percentage of charged groups in the membrane. Charged groups in the membranes increase osmotic pressure hence increasing water uptake [43]. As presented in Table 4, the **water uptake** of the membranes under study covered a wide range, from 0.21 g H_2_O/g dry AEM for the highly crosslinked FujiFilm-12, to 0.63 g H_2_O/g dry AEM for FujiFilm-10. However, since the dry weight of the AEMs includes the thick support of the Suez membranes, *WU* comparisons could be deceptive. To avoid this, the **hydration number** **λ** of the membranes was also calculated. This parameter shows that Neosepta AMX, FujiFilm-1, and FujiFilm-10 possess similar water content. FujiFilm-12 is the least hydrated, corresponding with its measured *WU*. Regarding the Suez membranes, their hydration numbers are higher than for the rest of the AEMs and similar between each other. This observation agrees with the hydration reported by the manufacturer, which, although given in % of wet resin, indicates that the membranes are similar.

#### 3.2.2. Electrochemical Characterization of Virgin Membranes

The **permselectivities** of the evaluated membranes were similar, around 0.9. Neosepta AMX presented the highest value, 0.94, consistent with previous reports in the literature [26,30]. The high permselectivity of the membranes was expected since this property is directly related to the fixed charge concentration, which is high for all the evaluated AEMs.

The **area resistances** of the membranes were measured in two different solutions: 0.5 M NaCl, which enables us to compare our measurements to others reported in the literature, and the BW solution employed during the desalination experiments.

Among the six AEMs, FujiFilm-12 exhibited the highest electrical resistance for both the NaCl solution (5.1 Ω·cm^2^) and the BW one (14.19 Ω·cm^2^). Despite FujiFilm-12 is a thin membrane, the high resistance was expected since this property is highly dependent on water content, which is itself linked to the fixed charge concentration of the polymer [39]. FujiFilm-12 presented a relatively low IEC, low hydration number, and, consequently, a high area resistance. Although much thicker than FujiFilm-12, the Suez membranes presented similar resistance values, which can be explained given their higher water content. The other two FujiFilm membranes (type 1 and type 10) show a low electrical resistance, as was expected considering their thin support and high-water content. The electrical resistance of Neosepta AMX was slightly higher than for FujiFilm-1 and FujiFilm-10, although they have similar thicknesses, which could be attributed to a higher crosslinking degree.

#### 3.2.3. Physico-Chemical Evaluation of Fouled Membranes

**Figure 6:**
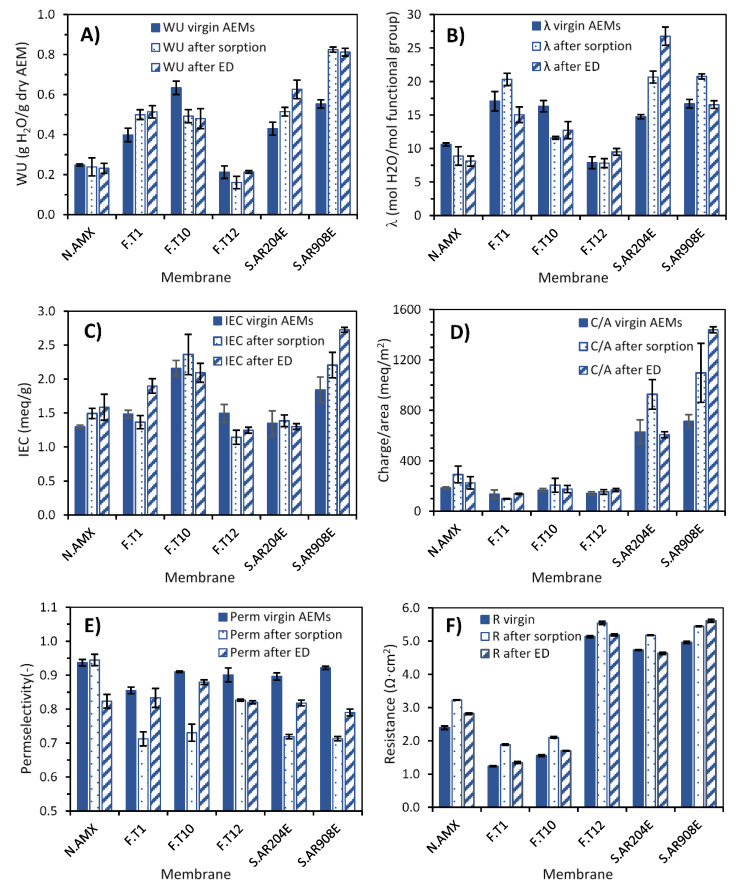
Comparison of membrane properties for virgin, soaked, and membranes fouled after ED. (**A**) Water uptake (WU), (**B**) hydration number (λ), (**C**) ion exchange capacity (IEC), (**D**) charge per area, (**E**) permselectivity, and (**F**) area resistance (in 0.5 M NaCl).

The **water uptake** (WU) measured for the AEMs is displayed in Figure 6A. Compared to the virgin membranes, the fouled membranes after sorption and after electrodialysis presented similar changes; that is, they both increased or decreased depending on the type of AEM. Most of the membranes presented a net increase in WU, and only Neosepta AMX and FujiFilm-10 membranes presented a decrease. The measured increases in WU are, at least partially, attributable to the presence of viscous solution being removed together with the AEM. The removal was evident because the fouled coupons had higher wet weights than the ones from virgin membranes, despite all being cut with the same surface dimensions. Although the surfaces of the coupons were smoothly cleaned with a tissue, it is likely that some HPAM-containing solution remained on the AEM, especially on those with a rougher surface.

If the comparisons are made in terms of the **hydration number**, as shown in Figure 6B, the results indicate that the thin membranes (Neosepta and the FujiFilm ones) presented a decrease in hydration. In contrast, for the Suez ones, the tendency is still an increase in hydration. The differences in hydration number could be due to some highly hydrated ions (calcium, magnesium, bicarbonate, and sulfate) remaining inside the pores of the thicker ion-exchange material, leading to stretching of the ion-exchange matrix and an increase in these pores [44]. The increase in pore size, as a rule, leads to an increase in the water content in the membrane, as well as to an increase in its diffusion permeability. Ghalloussi et al. also observed both increase and decrease of water content on aged AEMs [45].

Figure 6C shows the **IEC** measured for the virgin and fouled membranes. With a few exceptions, the IEC of the AEMs did not significantly change when fouled, either by sorption or by ED. The exceptions were FujiFilm-1 and Suez AR908E after ED, both of which presented a notorious increase of IEC compared to the virgin membranes. The increase in IEC could be caused by the presence of calcium ions, which could interact with the negatively charged HPAM to form a complex group with a positive net charge. The positive net charge group may provide extra charge carriers on the surface of the membranes hence increasing ion exchange capacity. Additional sorption tests were performed with only HPAM and NaCl solution (to exclude the effect of the divalent ions), and it was observed that the IEC of fouled AEMs dropped close to that of the virgin ones (data not shown).

The slight decreases in IEC are, on the contrary, commonly measured for aged or fouled membranes [45], and can have diverse causes. One is the foulant occupying the fixed charge group’s sites, thus reducing the number of fixed charge carriers. The reduction of IEC is also typical when the membranes were exposed to high pH, for example, when reaching limiting current conditions and hydroxide anion was formed. When operated under high pH conditions, the functional groups of AEMs can decompose, and its functional property is reduced [24,38].

#### 3.2.4. Electrochemical Characterization of Fouled Membranes

Figure 6E shows the **permselectivities** measured for all the AEMs. In this case, all fouled membranes presented the same behavior: their permselectivity decreased (except for Neosepta AMX after sorption, which remained the same). Two reasons can explain permselectivity reduction. First, HPAM and other charged molecules present in the solution probably interacted with the fixed charged groups of the AEMs and reduced their effective charge, therefore diminishing their ability to reject co-ions. A second reason is a permselectivity dependence on the binding affinity of the counterion with the polymer fixed charge groups [35]. Given the composition of the BW employed during the experiments, it is expected that varied counterions (Cl^−^, SO_4_^2−^, and HCO_3_^−^) would be present inside the AEM matrix, thus affecting membrane permselectivity.

Permselectivity also can be affected by the water content of the membrane, and the higher the water sorption, the lower the permselectivity of an AEM [39]. This relationship explains why the sharpest decreases in permselectivity (above 10%) were recorded for the Suez membranes, which presented the highest increase in water uptake (Figure 6A).

Furthermore, it is interesting that the permselectivity drops were more severe upon AEM sorption. Two factors might have influenced this result. First, the crude oil incorporated in the feed solutions from the last stages might foul the AEMs in a different form. Second, the dynamic operation in the ED-stack, particularly regarding fluid hydrodynamics, might have limited the amount of foulant remaining on the membranes.

Figure 6F shows the **electrical resistance**, measured in 0.5 M NaCl, of the six AEMs. For most of the fouled membranes, there was a slight increase after fouling from both sorption and ED process, and even in a few cases, the resistance of the fouled membranes did not have a significant change. The measured resistance depends on the exchange capacity, the quantity and volume fraction of meso- and macropores, as well as the resistance of the solutions filling these pores [46]. It must be recalled that the membrane coupons employed for resistance measurement were the same previously employed for measuring permselectivity (see Section 2.3.3), so any foulant that was not firmly attached was removed by the fluid while measuring permselectivity. Therefore, the increase in resistance could be attributed to some firmly attached foulants (HPAM, oil), which attach to the membrane and reduce the available surface area for ion permeation. Resistance can also be affected by the presence of varied counterions on the AEM matrix, which affect the water content of the membrane [39].

## 4. Discussion

Now we proceed to relate the performance of the stacks composed by different AEMs with the information gathered during the individual membrane characterization. Since the most considerable differences in stack performance were observed in terms of voltage increase, we first focused on explaining this behavior. To do so, we modeled the ED stack as an analogous DC circuit in which the different components (CEM, AEM, diluate, and concentrate compartments) are represented as a series of resistances, as explained in Section 2.3.8. The parameters employed in the model are summarized in Table 6.

**Figure 7:**
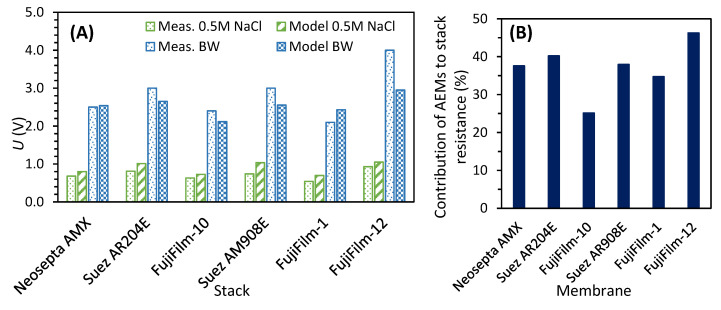
(**A**) Comparison of modeled and measured voltages for the different stacks operated at 100 A/m^2^. (**B**) Contribution of AEM resistance to the stack resistance according to the model for the BW solution.

Figure 7A shows that the modeled results are close to the ones measured during stage I, when a 0.5 M NaCl was desalinated (Table 4). Furthermore, the same figure includes the results obtained after adjusting the model to estimate the potential required to desalinate the BW solution, when the process was still running in under-limiting conditions. For that case, CEM and AEM resistances had to be considered, as indicated in Table 6. The voltages thereby obtained also approached the ones measured when the BW desalination was just started.

Besides reasonably predicting the voltages required by the different stacks, the model allowed us to weight the contribution of each of the cell pair’s components. Focusing on the AEM’s, we estimated that for the membrane with the lowest resistance, FujiFilm-10, the AEMs represent 26% of the total stack resistance. In contrast, for the stack composed by FujiFilm-12, the AEMs represent 48% of the resistance (Figure 7B).

Please note that the model is applied to the initial conditions of stage II, when only BW was being desalinated. However, not all stacks behaved the same during this stage. While for three of the stacks (Neosepta, FujiFilm-1, and FujiFilm-10), the applied voltage stabilizes very fast; for the other three membranes, it remains to fluctuate during the stage (see Figure 3). This difference could be attributed to the membranes still conditioning to the composition of the BW solution (given their thickness and high degree of crosslinking they take longer to do so), but also to the likely development of concentration polarization. This explains the differences between the modeled voltage values and the measured ones. Then, when the HPAM-containing solution is incorporated in stage III, concentration polarization increased and reduced the active area of the membranes, causing the surge in voltage. Figure 3 shows that all stacks were somehow sensitive to the incorporation of BW + P solution, but the ones containing AEMs with lower electric resistance had smaller changes than the others.

In this regard, it appears that the other membrane properties analyzed in this study cannot be so easily related to the development of concentration polarization. Figure 4 shows that lower desalination performances were recorded for the stacks composed by FujiFilm-12 and Suez AR908E. Both AEMs coincide in having high area resistance, but their other properties differ significantly. FujiFilm-12 has low WU, low IEC, and charge/area, while Suez AR908E combines a high WU with high IEC and charge/area.

**Figure 8:**
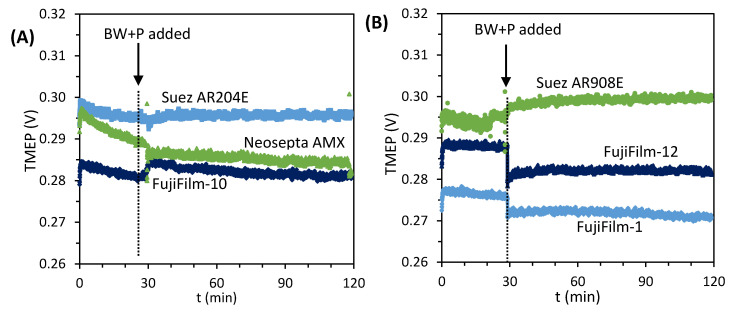
Measured transmembrane electric potential (TMEP) vs. time for fouling experiments in the six-compartment cell. BW was desalinated at 28 A/m^2^ for 30 min, after which the feed was substituted by the BW + P solution. Plot (**A**) presents results for Neosepta AMX, Suez AR204E, and FujiFilm-10, while plot (**B**) includes Suez AR908E, FujiFilm-1, and FujiFilm-12. The jumps in TMEP are caused by small variations in the conductivity of the solution.

Further tests were performed on single AEMs desalinating BW + P solutions at moderate current density (28 A/m^2^) in the six-compartment cell (Section 2.3.5). During the tests, the transmembrane electric potential (TMEP) was monitored continuously. Any sudden increase could be related to the development of concentration polarization and fouling on the different AEMs when exposed to the BW + P solution [17]. However, the results presented in Figure 8 indicated that desalinating the BW + P solution did not cause a notorious increase of the TMEP, independently of the AEM under observation. For example, the membranes with low water content (Neosepta AMX and FujiFilm-12), had similar behavior to those with high water content (Suez membranes). These results suggest that the high voltages achieved during the stack operation were not only driven by AEM fouling. Thus, it is likely that other factors were triggering the increase in voltage.

Then, it is interesting to pay attention to other phenomena happening in the cell, like concentration polarization and fouling of the spacer. The clean spacers already had a significant shadow factor, but this effect might have increased when they were exposed to HPAM-containing solution. The spacers, made of polyamide, had likely a high affinity with the polyacrylamide contained in the BW + P solution. Thus, the additive effects of higher solution viscosity plus the higher affinity with the spacer, the active area of the membranes in contact with the BW + P solution, was effectively reduced. For the stacks containing low-resistance membranes, this did not have a significant effect.

On the contrary, the stacks that contained AEMs with higher resistance reached LCD conditions almost immediately. According to the Nernst–Planck equation, higher resistance is due to lower diffusivity or thicker membrane. Ion transport by migration at high resistance can still be high by applying high voltage, but the driving force for diffusive ion transport cannot be compensated. Thus, the effect of high resistance is a change of transport mechanism, fewer diffusions, more migration. Then, the LCD occurs in the boundary layer next to the membrane due to the depletion of ions, causing water splitting phenomena and possibly damaging the AEMs due to the local increase of pH [24]. However, the pH meters placed after the ED cell registered relatively small changes (<1.0), probably due to the bicarbonate in solution acting as a pH buffer.

Regarding the influence of the membrane chemistry, its role probed not to be as critical as expected [47]. While desalinating the BW + HPAM solution, the aromatic (Neosepta AMX) and some of the aliphatic membranes had relatively stable performances. When adding crude oil to the feed and with it some aromatic compounds, the performance of all stacks stabilized (Figure 3). The pictures of the recovered AEMs (Figure S5) suggest similar degrees of fouling for all of them. It is also possible that surface properties which were not measured in this investigation, like the roughness and hydrophilicity of the AEM, influenced their performance in the stack. However, these effects might have been minimal, as suggested by the single membrane experiments in Figure 8.

Since the aim of this work was to compare the membranes after operating under similar conditions, the current density was maintained despite the high voltage, which could have affected the membranes. For other purposes, like operating the system during extended times, the current density should be adjusted according to the characteristics presented by each stack.

## 5. Conclusions

Six different ED stacks, each containing a different type of homogeneous AEM with quaternary ammonium groups, were employed during 8-day experiments to desalinate PFPW. During three of the six EDR runs, limiting conditions were reached, which was attributed to the combination of high intrinsic area resistance of some of the AEMs and the development of concentration polarization when HPAM-containing solution was fed to the stack. The incorporation of crude oil in the feed solution did not have a negative effect in terms of increasing the stack resistance, and, in some cases, it even caused its decrease and stabilization. Operating the electrodialysis in reverse mode was only beneficial during some minutes after the switch, after which the required voltage increased again due to concentration polarization.

This experimental work proved the close relationship between membrane properties. Changes in their physico-chemical characteristics, like water content and IEC, were also reflected in their electrochemical behavior, namely permselectivity and area resistance. Membrane properties measured after ED and after sorption experiments generally followed the same trends, suggesting that most changes were mainly due to processes occurring on the surface of the membranes and not due to their internal poisoning. When poisoning occurs, the volume, the water content, and the resistance increase [48]. For some membrane properties, the measured changes were more substantial for the AEMs soaked in BW + P solution (without oil) than for the AEMs recovered from the ED stack, which suggests that even small concentrations of oil in solution influences the interaction between AEMs and the surrounding solution.

Although all tested AEMs operated smoothly after the operating current density was decreased to 75 A/m^2^, this work evidenced the advantages of using membranes with low area resistance, which are operating at higher current densities and diminishing the risk of reaching limiting conditions. In that context, for future works, we would choose AEMs with a low resistance to desalinate PFPW. Since resistance is a consequence of material characteristics, the desired membrane would need to be relatively thin, have high IEC, and moderate to high water content. However, factors like mechanical and chemical stability, which were not analyzed in this investigation, could turn out to be more favorable for the reinforced membranes. Thus, for future studies, it would be desirable to (1) evaluate the AEMs performance for reaching the final desalination objective, and (2) assess the long-term stability of different kinds of AEMs.

## Figures and Tables

**Table 1:** Ionic composition of synthetic brackish water (BW) solution.

Ion	Concentration (mM)
Na^+^	73.92
K^+^	0.72
Ca^2+^	0.65
Mg^2+^	0.46
Cl^−^	56.24
HCO_3_^−^	15.59
SO_4_^2−^	2.51

**Table 2:** Summary of chemical and physical properties of the six anion exchange membranes (AEMs) investigated as reported by the manufacturers.

AEM Type.	Thickness (µm)	Area Resistance * (Ω·cm^2^)	Chemistry	Description	Application
Neosepta AMX	135	2.4	Styrene-divinyl benzene	High crosslinking, high mechanical strength	Desalination of food, concentration of inorganic salt, hardness removal
FujiFilm-1	110	0.9	3D-structure of inert polyolefin fibers filled with aliphatic polyamide [32]	Low power consumption and medium water permeating	Water softening by capacitive deionization
FujiFilm-10	130	1.6	3D-structure of inert polyolefin fibers filled with ion-exchange material [32]	Low power consumption and medium water permeating	Purifying process/wastewater/brackish water/food streams by ED
FujiFilm-12	130	1.7	Same chemistry as FujiFilm-10, but different support	-	-
Suez AR204E	550	5.5	Copolymers of vinyl monomers	Homogeneous ion-exchange film. Long-term stability and high resistance to fouling by organic materials	Water treatment
Suez AR908E	650	6.0	Copolymers of vinyl monomers	Homogeneous ion-exchange film. Long-term stability and high resistance to fouling by organic materials	Wastewater treatment

* Measured in 0.5 M NaCl.

**Table 3:** Feed compositions, current density, flow rate, velocity, and times of the different experimental stages.

Stage	Feed Solution (10 L Volume)	Current Density (A/m^2^)	Flow Rate (mL/min)	Fluid Velocity (cm/s)	Time (h)
I	0.5 M NaCl	100	150	1.6	Overnight
II	BW	100	150	1.6	6
III	BW + 50 mg/L HPAM	100	180	1.9	24
IV	BW + 100 mg/L HPAM	100	210	2.2	24
V	BW + 200 mg/L HPAM	75	240	2.5	24
VI	BW + 200 mg/L HPAM + 20 mg/L oil	75	240	2.5	48
VII	BW + 200 mg/L HPAM + 40 mg/L oil	75	240	2.5	60
VIII	BW + 200 mg/L HPAM + 40 mg/L oil	100	240	2.5	14

**Table 4:** Average absolute potential |*Ū*| measured while desalting NaCl 0.5 M solution at 100 A/m^2^.

Stack	|*Ū*| (V) Stage A (0.5 M NaCl)
Neosepta AMX	0.68
Suez AR204E	0.81
FujiFilm-10	0.63
Suez AR908E	0.74
FujiFilm-1	0.54
FujiFilm-12	0.93

**Table 5:** Values measured and reported in the literature for membrane properties. Typical repeat errors for permselectivity are below 0.02.

AEM Type	Wet Thickness (µm)	IEC (meq/g Dry AEM)	Water Uptake (g H_2_O/g dry AEM)	Hydration Number λ (mmol H_2_O/meq)	Perm-Selectivity (−)	Area Resistance in 0.5 M NaCl (Ω.cm^2^)	Area Resistance in BW (Ω.cm^2^)
Neosepta AMX	137 ± 2 141 [26], 138 [30]	1.3 ± 0.03 1.4 [26], 1.3 [30]	0.25 ± 0.02 0.23 [26], 0.16 [30]	10.6 ± 0.9	0.94 0.94 [26], 0.91 [30]	2.39 ± 0.05 2.77 [26], 2.35 [30]	9.92 ± 0.14
FujiFilm-1	127 ± 2 139 [25]	1.49 ± 0.24 1.8 [26]	0.40 ± 0.03 0.56 [26]	17.1 ± 1.4	0.86 0.90 [26]	1.24 ± 0.01 1.05 [26]	8.77 ± 0.11
FujiFilm-10	151 ± 3	2.16 ± 0.12	0.63 ± 0.03	16.3 ± 0.8	0.91	1.56 ± 0.02	5.54 ± 0.12
FujiFilm-12	148 ± 13	1.49 ± 0.18	0.21 ± 0.02	7.9 ± 0.23	0.90	5.13 ± 0.03	14.19 ± 0.26
Suez AR204E	616 ± 11	1.35 ± 0.18 2.34 ^a^	0.50 ± 0.03	14.8 ± 0.3 0.42 ^b^	0.90	4.73 ± 0.01	11.09 ± 0.18
Suez AR908E	655 ± 14	1.84 ± 0.19 1.97 ^a^	0.55 ± 0.04	16.7 ± 0.9 0.43 ^b^	0.92	4.96 ± 0.03	10.08 ± 0.19

^a^ The manufacturer reports the IEC as meq/g of dry resin, so the values cannot be compared directly. ^b^ Values reported by the manufacturer as % of the wet resin.

**Table 6:** Parameters used for the ED model.

Parameter	Value
CEM resistance (0.5 M NaCl)	3.3 Ω·cm^2^ [40]
CEM resistance (BW)	7.0 Ω·cm^2^ [40]
AEM resistance (0.5 M NaCl and BW)	Table 5
Shadow factor *β*	0.48 [41]
Spacer volume *V_sp_*	0.2
Current density	100 A/m^2^

## Supplementary Materials

The following are available online at https://www.mdpi.com/2077-0375/10/11/352/s1, Figure S1. Scheme of the two-compartment cell utilized for potential measurement; Figure S2. Calibration curve relating the HPAM concentration to the measured TOC; Figure S3. Electric potential *U* and current density *i* versus time during the desalination of NaCl 0.5 M; Figure S4. Current density vs. voltage curves obtained when desalinating BW solution in stacks; Figure S5. Photographs of anion exchange membranes recovered after the EDR runs.
