# Supplementary material for: Experimental Evaluation of Anion Exchange Membranes for the Desalination of (Waste) Water Produced after Polymer-Flooding
Source: Membranes (Basel). 2020 Nov 18;10(11):352. doi: 10.3390/membranes10110352 (PMC7698788; doi:10.3390/membranes10110352)
Supplement: Supplementary file 1 [file membranes-10-00352-s001.pdf]

Supplementary Materials

Experimental Evaluation of Anion Exchange Membranes for the Desalination of (Waste)Water Produced after Polymer-Flooding

Paulina A. Sosa-Fernández [1,2], Jan W. Post [1], Harrison L. Nabaala [1], Harry Bruning [2,*] and Huub Rijnaarts [2]

[1] Wetsus, European Centre of Excellence for Sustainable Water Technology, P.O. Box 1113, 8911CC Leeuwarden, The Netherlands; Paulina.sosafernandez@wetsus.nl (P.A.S.-F.); jan.post@wetsus.nl (J.W.P.); nleshao88@gmail.com (H.L.N.)

[2] Wageningen University, Department of Environmental Technology, P.O. Box 8129, 6700 EV Wageningen, The Netherlands; huub.rijnaarts@wur.nl

* Correspondence: harry.bruning@wur.nl; Tel.: +31-(0)31-748-3798

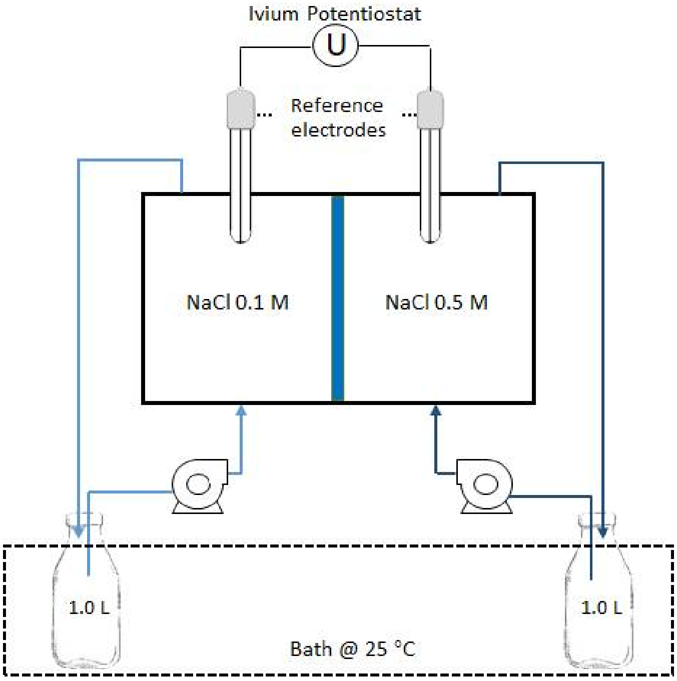

Figure S1. Scheme of the two-compartment cell utilized for potential measurement, the potentiostat measures the open cell voltage. The solutions were stored in a warm bath at 25 °C.

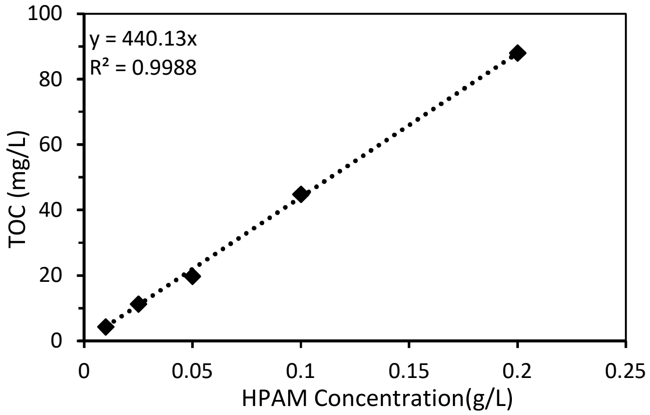

Figure S2. Calibration curve relating the HPAM concentration to the measured TOC.

Additional information for Results and discussion

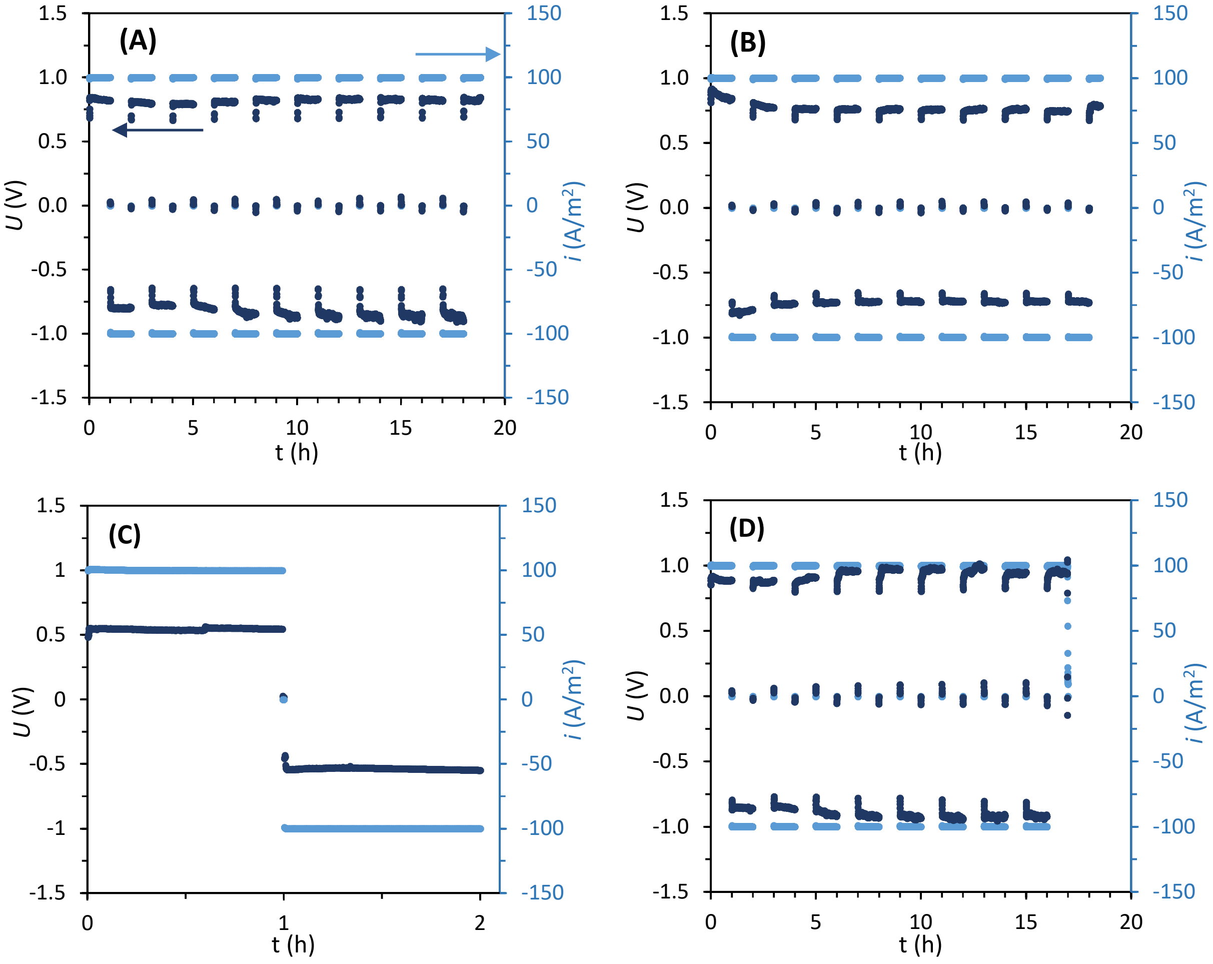

Figure S3. Electric potential *U* (marine) and current density *i* (light blue) versus time during the desalination of NaCl 0.5M (stage A) in the EDR mode (continuation of Figure 2). Values measured for the stack composed by **(A)** Suez AR204E, **(B)** Suez AR908E, **(C)** FujiFilm-1 (incomplete data set due to a failure in the recording system), and **(D)** FujiFilm-12.

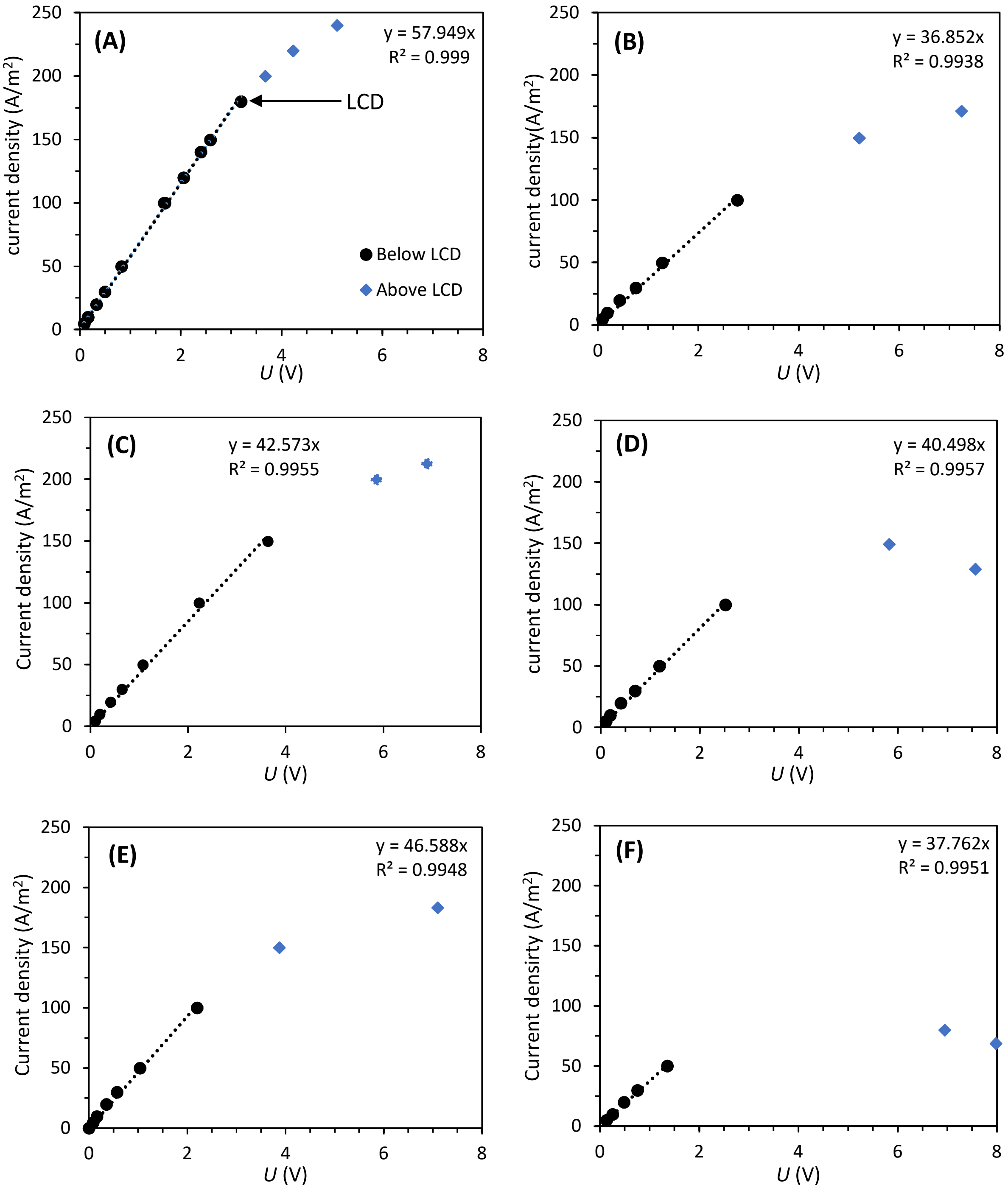

Figure S4. Current density vs. voltage curves obtained when desalinating BW solution in stacks containing **(A)** Neosepta AMX, **(B)** Suez AR204E, **(C)** FujiFilm-10, **(D)** Suez AR908E, **(E)** FujiFilm-1, and **(F)** FujiFilm-12. For Neosepta AMX, the LCD was calculated, while for the other AEMs, the test was only performed until reaching 100 A/m^2, to make sure that the operation would remain in the ohmic regime (linear trend).

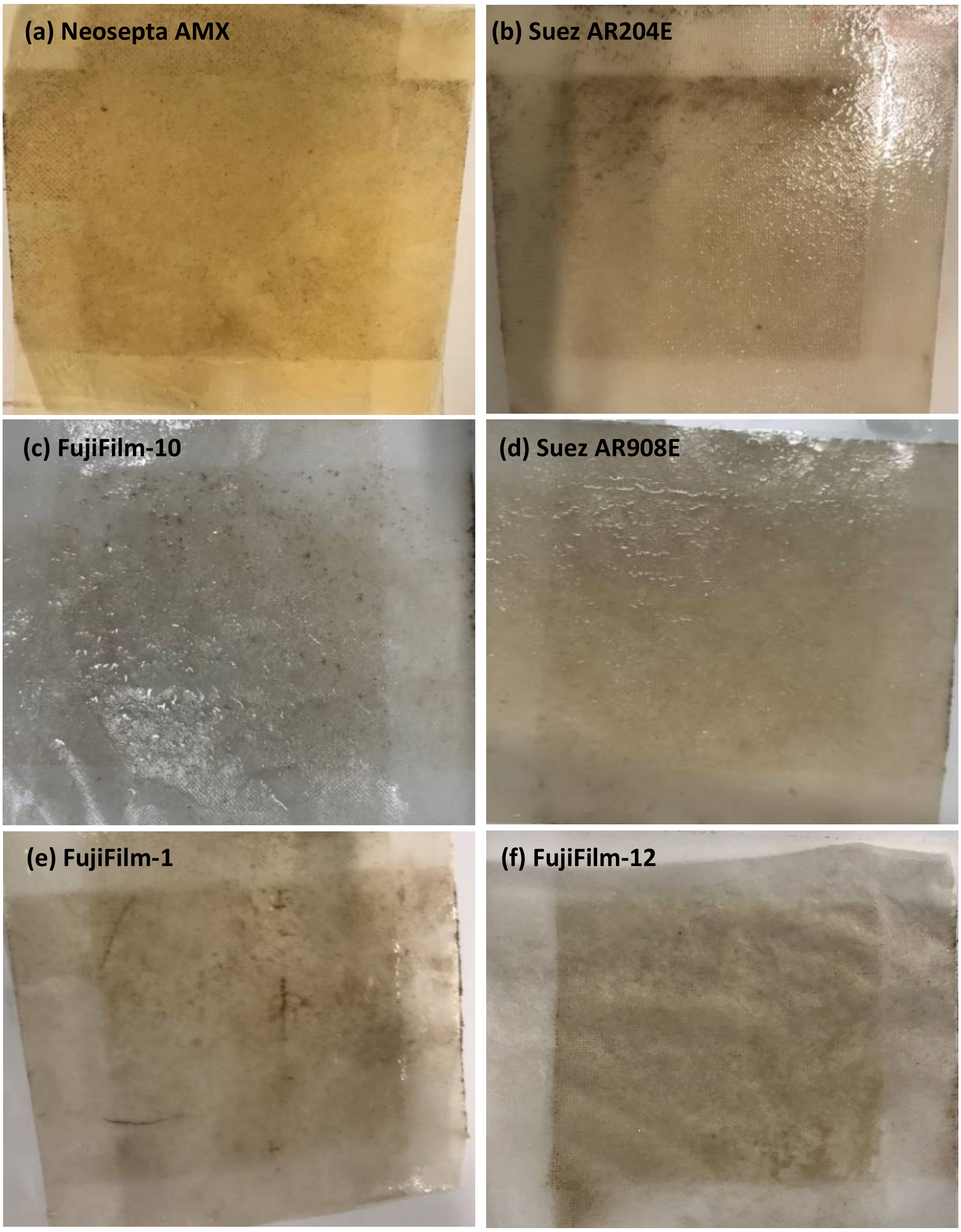

Figure S5. Photography's of anion exchange membranes recovered after the EDR runs
